# Outcomes with Avelumab Maintenance Treatment for Advanced Urothelial Cancer in a US Patient Cohort

**DOI:** 10.3390/curroncol33030138

**Published:** 2026-02-27

**Authors:** Kenneth Carson, Seyed Hamidreza Mahmoudpour, Chiemeka Ike, Sebastian Monzon, Stamatina Fragkogianni, Mairead Kearney

**Affiliations:** 1 Department of Internal Medicine, Division of Hematology/Oncology, Northwestern University Feinberg School of Medicine, Arkus Professional Building, 676 N. St. Clair St., Suite 850, Chicago, IL 60611, USA; 2 Merck Healthcare KGaA, 64293 Darmstadt, Germany; 3 EMD Serono, Inc., 200 Pier 4 Blvd., Boston, MA 02210, USA, an affiliate of Merck KGaA; 4 Tempus AI, Inc., 600 W Chicago Ave., Suite 510, Chicago, IL 60654, USA

**Keywords:** avelumab, carcinoma, real-world studies, urinary bladder neoplasms

## Abstract

Background: This study describes treatment patterns and clinical outcomes in patients with advanced urothelial carcinoma (aUC) in the US following the approval of avelumab for first-line maintenance treatment. Methods: This retrospective cohort study used deidentified patient data from the Tempus database. Eligible patients had completed first-line systemic anticancer treatment for aUC between July 2020 and March 2023. Results: In total, 974 eligible patients were identified; most (72%) were male. Median age at diagnosis was 70 years. Among patients who completed first-line platinum-based chemotherapy (644 [66%]), 574 (89%) had no evidence of disease progression. Of 219 patients who received first-line maintenance, 135 (62%) received avelumab. Median (95% CI) overall survival (OS) and progression-free survival (PFS) from avelumab maintenance start were 14.9 months (13.1—not estimable [NE) and 6.4 months (4.6—NE), respectively. Enfortumab vedotin (EV) was the most common second-line treatment after avelumab (70%). Median (95% CI) OS and PFS from second-line EV start were 11.6 months (6.1—NE) and 6.6 months (4.1—NE), respectively. Conclusions: Results provide insights into the impact of avelumab first-line maintenance treatment in patients with aUC in the US. Effectiveness data are consistent with previous findings, supporting the use of avelumab maintenance in patients without disease progression following first-line platinum-based chemotherapy. Second-line EV after progression on avelumab maintenance had similar effectiveness to results from other real-world studies.

## Simple Summary

In this study, we looked at medical records showing treatment patterns and outcomes in people with advanced urothelial cancer in the United States following the approval of avelumab for first-line maintenance treatment. Most people who completed first-line treatment received platinum-based chemotherapy (66%), and 89% of these people had no evidence of disease progression. Most people who went on to receive first-line maintenance received avelumab (62%). Outcomes with avelumab maintenance were similar to previous studies. Our study also demonstrates that, after disease progression on avelumab maintenance, second-line enfortumab vedotin appears to be an effective treatment option. Overall, available data support the use of avelumab maintenance as a standard treatment in people whose disease has not progressed following first-line platinum-based chemotherapy. Further research is needed to look at additional available treatment options and their associated outcomes when received outside of a clinical trial.

## 1. Introduction

Bladder cancer is the sixth most common cancer in the US, with 82,290 new cases and 16,710 deaths recorded in 2023 [1]. Age-adjusted incidence and mortality rates for bladder cancer in the US are 18.2 and 4.2 per 100,000 per year, respectively. Patients with advanced bladder cancer have a poor prognosis, with a 5-year survival rate of 8.3% for patients diagnosed with metastatic disease [2]. Over 90% of bladder cancer cases are caused by urothelial carcinoma (UC), and UC can also arise in the renal pelvis, ureter, and urethra [3].

Platinum-based chemotherapy (PBC) has been an established first-line (1L) treatment for advanced UC (aUC) for decades, with cisplatin- or carboplatin-containing combinations recommended for cisplatin-eligible or -ineligible patients, respectively. Although PBC is associated with an objective response or stable disease in 72% to 79% of patients, long-term benefits are limited, and median progression-free survival (PFS) and overall survival (OS) with PBC alone are only 6 to 7 months and 12 to 14 months, respectively [4,5,6]. In 2020, avelumab (immune checkpoint inhibitor [ICI]) administered as maintenance treatment became the standard treatment for patients with aUC who are progression-free following PBC based on the results of the JAVELIN Bladder 100 phase 3 trial [3,7]. After ≥2 years of follow-up in all patients, median OS from the start of avelumab 1L maintenance plus best supportive care vs. best supportive care alone was 23.8 vs. 15.0 months (hazard ratio, 0.76 [95% CI, 0.63–0.91]; *p* = 0.0036), and median PFS was 5.5 vs. 2.1 months (hazard ratio, 0.54 [95% CI, 0.46–0.64]; *p* < 0.0001), respectively [8]. In December 2023 and March 2024, respectively, enfortumab vedotin (EV) plus pembrolizumab and nivolumab plus cisplatin/gemcitabine (in cisplatin-eligible patients) were approved in the US for 1L treatment of aUC [3,9,10,11,12]. ICI monotherapy is also an approved 1L treatment option for cisplatin-ineligible patients with PD-L1–positive tumors and for platinum-ineligible patients; however, this approach is not considered a preferred treatment in recently updated guidelines [3].

Given the rapidly evolving treatment landscape in aUC and the need to understand the effectiveness of new treatment options after clinical adoption, it is important to assess the impact of new treatments in the real-world setting. Herein, we describe real-world patient characteristics, treatment patterns, and clinical outcomes in patients with aUC in the US after avelumab maintenance treatment was approved in June 2020. This analysis occurred before the approval of EV plus pembrolizumab and nivolumab plus cisplatin-based chemotherapy.

## 2. Materials and Methods

### 2.1. Study Design and Patients

This study was a retrospective analysis of data from the Tempus database, comprising longitudinal structured and unstructured deidentified data from geographically diverse oncology practices, including integrated delivery networks, academic institutions, and community practices. Database reliability and robustness were maintained with a rigorous data quality framework based on the Observational Health Data Sciences and Informatics (OHDSI) standard. This standard evaluates data across four key areas: Conformance, Completeness, Plausibility, and Drift. Our comprehensive approach utilized approximately 1150 checks throughout the Tempus data pipeline, from initial clinical data curation and sequencing to final data harmonization and delivery. This multi-layered system, including validation gates, downstream generic checks, and over 150 terminal logical checks, ensures that these real-world data are of high quality and reliability for a broad range of analytic use cases. Additionally, the database contains a higher proportion of late-stage patients compared to the entire population of patients with UC [13,14,15,16]. Eligible patients were diagnosed with locally advanced or metastatic UC (defined as ≥1 of the following: T4b, N2, N3, M1, or overall cancer stage 3 or 4) between January 2016 and March 2023 and had received ≥1 dose of prior systemic therapy. Patients with metastatic relapse were also included. Patients who completed 1L treatment on or after 1 July 2020 (i.e., after the US approval of avelumab 1L maintenance in June 2020) were analyzed in detail. The data cutoff was 29 March 2023.

### 2.2. Data Analysis

Data were obtained from 3 sources: clinical–genomic records, clinical records, and electronic medical records. Patient demographics on the date of diagnosis of aUC were recorded from patient charts. Response outcomes were derived from patient notes using general guidelines shown in Supplementary Table S1. Evaluated real-world outcomes included OS (primary outcome; defined as the time from treatment initiation until death from any cause), PFS (secondary outcome; defined as the time from treatment initiation until first documentation of progressive disease or death from any cause), and time on treatment (defined as the time between the first and last dose of a specific treatment, if available, or until the initiation of a new anticancer treatment). In separate analyses, treatment initiation referred to 1L PBC, avelumab 1L maintenance, or second-line (2L) treatment, as specified in the Results section. For PFS analysis, only progression events occurring ≥15 days after treatment initiation were considered, and the start of a subsequent line of treatment was considered as a proxy for progression and treated as a progression event. Progressive disease was defined as an increase in visible disease, an increase in the extent of disease, or the identification of new lesions. Progression events were documented either pathologically or radiologically and dictated by the physician. Curated dates associated with progression events were used in the PFS analysis. Reasons for discontinuation of avelumab 1L maintenance were obtained from unstructured clinical data, when available.

**Figure 1:**
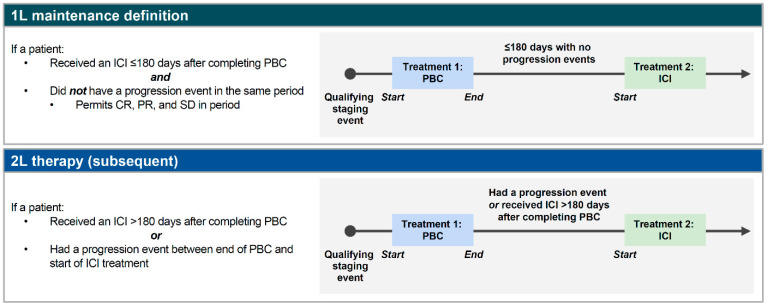
The algorithm used to categorize patients into 1L maintenance and 2L treatment subgroups. 1L, first line; 2L, second line; CR, complete response; ICI, immune checkpoint inhibitor; PBC, platinum-based chemotherapy; PR, partial response; SD, stable disease.

Patients who received subsequent treatment were categorized into subgroups (1L maintenance or 2L treatment) based on recorded clinical intent or algorithmically (i.e., 1L maintenance was defined as receipt of ICI treatment ≤ 180 days after completion of 1L PBC without progression [based on expert opinion of a practicing US clinical oncologist]; 2L treatment was defined as progression or receipt of ICI treatment > 180 days after completion of 1L PBC; Figure 1).

### 2.3. Statistical Analysis

Patient demographics and treatment patterns were summarized using descriptive statistics. Time-to-event outcomes (OS, PFS, and time on treatment) were estimated using the Kaplan–Meier method, and significance was determined using the log-rank method (*p* < 0.05). Follow-up was calculated using the reverse Kaplan–Meier method. All analyses were performed in R (version 4.2.3).

### 2.4. Ethics Approval

All patient-level data were deidentified in accordance with the Health Insurance Portability and Accountability Act. Tempus AI, Inc., has been granted an institutional review board exemption (Advarra Pro00072742) permitting the use of deidentified clinical, molecular, and multimodal data in order to derive or capture results, insights, or discoveries. This study followed the Strengthening the Reporting of Observational Studies in Epidemiology (STROBE) reporting guidelines [17].

## 3. Results

### 3.1. Patient Demographics and Treatment Patterns

Of 3299 evaluable patients with advanced bladder cancer who were identified in the database, 1939 (59%) received 1L systemic anticancer treatment, including 974 who completed 1L treatment after the approval of avelumab 1L maintenance (from 1 July 2020 onward; Supplementary Figure S1a). Within this postapproval population (Table 1), the majority of patients were male (*n* = 702 [72%]) and White (*n* = 588 [60%]), and the median age at diagnosis was 70 years (interquartile range, 63–77 years). Most patients were diagnosed with transitional cell carcinoma/UC (*n* = 842 [86%]). Of the 626 patients with an unblinded data source, 297 (47%) were identified as originating from academic institutions, 119 (19%) from community centers, and 210 (34%) from the American Society of Clinical Oncology CancerLinQ.

**Figure 2:**
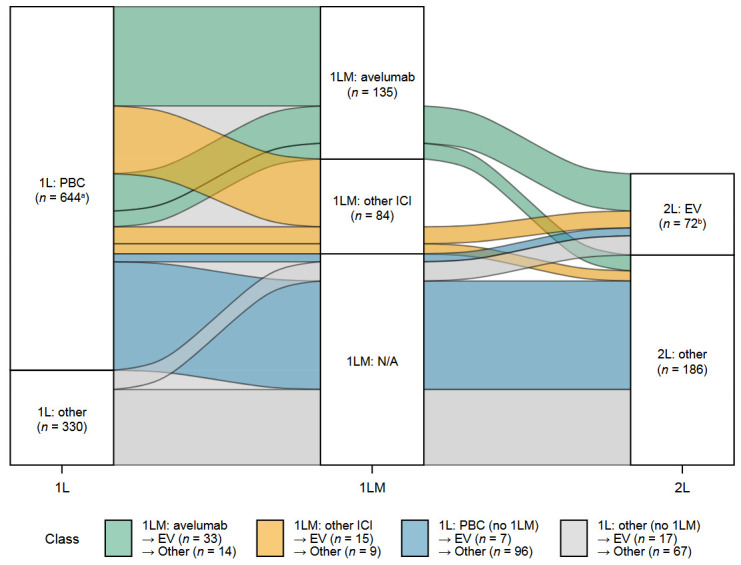
Treatment sequencing in patients who completed 1L treatment from July 2020 onward (post-approval population). 1L, first line; 1LM, first-line maintenance; 2L, second line; EV, enfortumab vedotin; ICI, immune checkpoint inhibitor; PBC, platinum-based chemotherapy. ^a^ Patients who did not receive any subsequent treatment are not represented by alluvia. ^b^ Of 72 patients who received 2L EV, 70 patients received EV monotherapy, 1 patient received EV plus paclitaxel, and 1 patient received EV plus an investigational drug.

The most common treatment regimens by treatment setting are shown in Supplementary Table S2. PBC was the most common 1L treatment (*n* = 644 [66%]). Among PBC-treated patients, 391 (61%) received a cisplatin-based regimen, 226 (35%) received a carboplatin-based regimen, and 19 (3%) received both cisplatin and carboplatin (Supplementary Figure S1b). After completing 1L PBC, 574 patients (89%) had no documented evidence of disease progression. Of the 219 patients who received 1L maintenance treatment (38% of the 574 patients without progression), 135 received avelumab (this was 62% of patients who received any 1L maintenance treatment and 24% of all patients without disease progression). Of 974 patients who completed 1L treatment, 258 (26%) received 2L treatment, most commonly EV (*n* = 72 [28%]). Of the 135 patients who received avelumab maintenance, 47 (35%) received 2L therapy, most commonly EV (*n* = 33 [70%]) (Figure 2).

### 3.2. Clinical Outcomes

In patients who received 1L PBC (*n* = 644), median follow-up from the start of 1L PBC was 10.2 months. Median time on 1L PBC treatment was 2.7 months (95% CI, 2.5–3.0 months). In this population, median OS from the start of 1L PBC was 13.6 months (95% CI, 12.1–17.8 months); the OS rate at 6 months from start of 1L PBC was 82% (95% CI, 78–87%) and at 12 months was 56% (95% CI, 50–63%) (Table 2). Median PFS from the start of 1L PBC was 3.5 months (95% CI, 3.3–4.1 months), and 6-month and 12-month PFS rates were 10% (95% CI, 6–17%) and 2% (95% CI, 1–7%), respectively.

**Figure 3:**
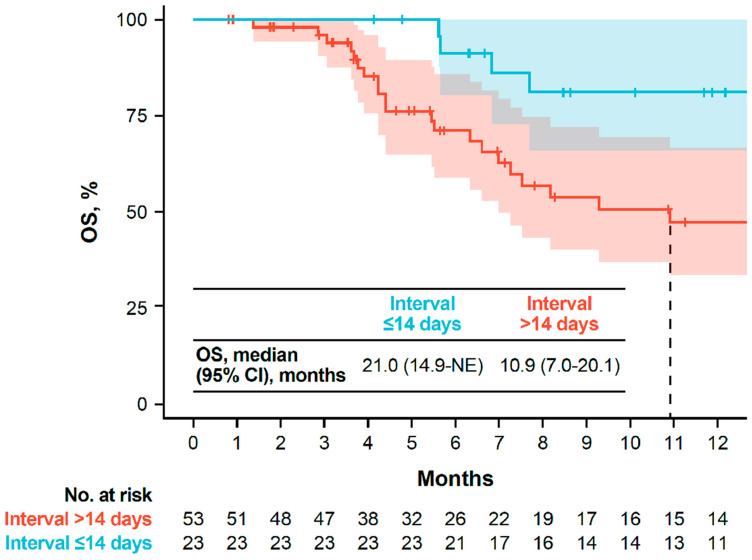
OS from the start of avelumab 1L maintenance in subgroups defined by the interval between the end of 1L PBC and the start of avelumab. 1L, first line; NE, not estimable; OS, overall survival; PBC, platinum-based chemotherapy.

Overall, 135 patients received avelumab 1L maintenance. In these patients, the median follow-up from avelumab maintenance initiation was 8.9 months, and the median time on avelumab maintenance treatment was 3.9 months (95% CI, 2.8–5.0 months). The median OS from the start of avelumab maintenance treatment was 14.9 months (95% CI, 13.1—not estimable [NE]), and 6- and 12-month OS rates were 80% (95% CI, 72–90%) and 63% (95% CI, 52–75%), respectively (Table 2). The median PFS from the start of avelumab maintenance was 6.4 months (95% CI, 4.6 months—NE), and 6- and 12-month PFS rates were 52% (95% CI, 42–65%) and 40% (95% CI, 30–54%), respectively. In patients with data available for the treatment-free interval from the end of 1L PBC to the initiation of avelumab maintenance, the median OS from the start of avelumab maintenance in subgroups that started avelumab ≤14 days (*n* = 23) or >14 days (*n* = 53) after the end of 1L PBC was 21.0 months (95% CI, 14.9 months—NE) and 10.9 months (95% CI, 7.0–20.1 months), respectively (Figure 3).

In patients who received 2L EV after avelumab 1L maintenance (*n* = 33), the median OS and PFS from the initiation of 2L EV were 11.6 months (95% CI, 6.1 months—NE) and 6.6 months (95% CI, 4.1 months—NE), respectively (Supplementary Figure S2).

## 4. Discussion

Our findings provide several insights into real-world treatment patterns and clinical outcomes in this population of patients with aUC in the period immediately following the approval of avelumab 1L maintenance treatment in the US. Consistent with guidelines from the study period, the most common 1L treatment received was PBC. Of patients without disease progression following 1L PBC, 38% received any 1L maintenance treatment, and 24% received avelumab, consistent with data reported from a previous analysis in a different US cohort [18]. These data suggest that many potentially eligible patients (i.e., those without disease progression after 1L PBC) did not benefit from the established efficacy of avelumab maintenance treatment. This result is consistent with the prior literature demonstrating uneven adoption of newly approved therapies in oncology practice [19]. Among patients who completed 1L treatment, only 26% had evidence of 2L treatment. The high level of attrition between 1L and 2L treatment in patients with aUC has been observed in various observational studies [20] and may reflect the rapid clinical deterioration that often accompanies disease progression in patients with aUC. Alternatively, this result could represent a change in care facility or incomplete data within the real-world data source. Of note, in the subgroup who received avelumab maintenance, a higher percentage of patients received 2L therapy (35%), indicating that avelumab maintenance resulted in lower levels of attrition.

In this study, patients who received avelumab 1L maintenance had a 12-month OS rate of 63% and a 12-month PFS rate of 40%. This observation is comparable to results of the JAVELIN Bladder 100 phase 3 trial, which reported 12-month OS and PFS rates of 71% and 30%, respectively [7,21]. Similarly, in other real-world studies of avelumab maintenance performed in different countries, 12-month OS and PFS rates ranged from 65% to 76% and 33% to 39%, respectively [22,23,24,25]. Thus, several real-world studies have demonstrated the effectiveness of avelumab maintenance in routine clinical practice, despite the real-world populations being more heterogeneous than the clinical trial population. In our study, longer OS was observed in patients whose treatment-free interval (defined as the time between the end of 1L PBC and start of avelumab maintenance) was ≤14 days vs. >14 days; however, these results should be interpreted with caution, given the small number of patients and potential differences in patient characteristics between subgroups. In the JAVELIN Bladder 100 trial, all patients had a treatment-free interval of 4 to 10 weeks, and trial outcomes were consistent between subgroups who had differing treatment-free intervals within this range [26]. These data suggest that additional analyses of outcomes in real-world patients with shorter treatment-free intervals may be informative.

In our study, EV was the most common 2L treatment after avelumab 1L maintenance (*n* = 33; 70% of patients who received 2L treatment). Median OS and PFS from the start of 2L EV treatment in these patients were 11.6 and 6.6 months, respectively. Although analyses in this small population should be interpreted with caution, these findings appear similar to data for EV treatment in patients who had received 1L PBC and had subsequent progression during or after 2L ICI treatment in the phase 3 EV-301 trial (median OS, 12.88 months; median PFS, 5.55 months) [27]. Similar outcomes have been seen in other real-world studies of 2L EV received after avelumab maintenance treatment [28,29,30]. In the AVENANCE real-world study of avelumab maintenance performed in France, median OS from the start of avelumab in the subgroup who received 2L antibody–drug conjugate treatment (mostly EV) was 31.3 months, compared with 14.4 months in patients who received 2L chemotherapy [31]. Following the results of the EV-302 phase 3 trial [12], an increasing proportion of patients are expected to receive 1L EV plus pembrolizumab. However, evidence is limited to inform the optimal treatment sequence for patients with aUC, considering efficacy, safety, quality of life, and costs of all available treatment options across different lines [32]. Findings from our study and others suggest that for patients who have received the JAVELIN Bladder regimen of 1L PBC and avelumab maintenance in those without progressive disease, 2L EV remains an effective treatment sequencing option. 

The study adds to the growing body of real-world evidence for avelumab 1L maintenance treatment. It is differentiated from other real-world studies by reporting detailed data from diverse oncology practices across the US in a time period immediately following the FDA approval of avelumab maintenance, in addition to providing insights about patient characteristics and outcomes in those who received 1L PBC, avelumab maintenance, and 2L treatment after avelumab. However, the study has several limitations that should be considered when interpreting its findings. Analyses were performed using the Tempus database, which includes data from various sources captured during routine clinical care; thus, the data obtained may be incomplete or underreported in some patients. No external chart review validation was performed, and as such, the study’s findings are exploratory. Definitions of 1L maintenance and 2L treatment may differ between our study and clinical practice, and because algorithms for lines of treatment were based on assumptions, they may have resulted in misclassifications and may not accurately reflect the clinical definition of a new line of treatment. Furthermore, median follow-up time from the start of avelumab maintenance was short; therefore, some patients may have had insufficient follow-up to observe treatment patterns beyond 1L treatment or to accurately estimate survival outcomes.

## 5. Conclusions

This real-world study reveals treatment patterns and outcomes in patients with aUC in the US after the approval of avelumab 1L maintenance but before the approval of 1L EV plus pembrolizumab and 1L nivolumab plus cisplatin-based chemotherapy. Early uptake of avelumab maintenance was observed. Clinical outcomes observed support the use of avelumab maintenance treatment for patients with aUC who are progression-free following 1L PBC. Additional studies are needed to confirm the generalizability of results from clinical trials in the diverse real-world aUC population. Following the approval of additional 1L treatment options since this study was conducted, future research should evaluate optimal treatment sequencing and associated clinical outcomes in patients with aUC.

## Figures and Tables

**Table 1:** Patient demographics in subgroups of patients who completed 1L treatment from July 2020 onward (postapproval population).

Parameter	Received Any 1L Treatment (*n* = 974)	Received 1L PBC (*n* = 644)	Received Avelumab 1L Maintenance (*n* = 135)	2L EV After Avelumab 1L Maintenance (*n* = 33)
Age at aUC diagnosis, median (IQR), years	70 (63–77)	68 (61–75)	69 (63–75)	69 (61–75)
Unknown, *n*	72	48	9	2
Year of aUC diagnosis, *n* (%)				
2016	13 (1.3)	8 (1.2)	NA	NA
2017	10 (1.0)	6 (0.9)	NA	NA
2018	11 (1.1)	8 (1.2)	1 (0.7)	NA
2019	59 (6.1)	39 (6.1)	2 (1.5)	NA
2020	285 (29.3)	197 (30.6)	45 (33.3)	17 (51.5)
2021	399 (41.0)	261 (40.5)	73 (54.1)	14 (42.4)
2022	190 (19.5)	119 (18.5)	14 (10.4)	2 (6.1)
2023	7 (0.7)	6 (0.9)	NA	NA
Sex, *n* (%)				
Female	272 (27.9)	184 (28.6)	31 (23.0)	11 (33.3)
Male	702 (72.1)	460 (71.4)	104 (77.0)	22 (66.7)
Race, *n* (%)				
American Indian or Alaska Native	5 (0.5)	3 (0.5)	1 (0.7)	NA
Asian	19 (2.0)	15 (2.3)	1 (0.7)	1 (3.0)
Black or African American	44 (4.5)	28 (4.3)	4 (3.0)	1 (3.0)
Native Hawaiian or other Pacific Islander	1 (0.1)	1 (0.2)	NA	NA
Other	29 (3.0)	22 (3.4)	5 (3.7)	2 (6.1)
Unknown	288 (29.6)	199 (30.9)	36 (26.7)	10 (30.3)
White	588 (60.4)	376 (58.4)	88 (65.2)	19 (57.6)
Region, *n* (%)	*n* = 344	*n* = 219	*n* = 55	*n* = 14
Midwest	164 (47.7)	95 (43.4)	25 (45.5)	5 (35.7)
Northeast	32 (9.3)	21 (9.6)	7 (12.7)	2 (14.3)
South	78 (22.7)	54 (24.7)	13 (23.6)	4 (28.6)
West	70 (20.3)	49 (22.4)	10 (18.2)	3 (21.4)
Histology type, *n* (%)				
Ambiguous carcinoma	90 (9.2)	68 (10.6)	9 (6.7)	3 (9.1)
Transitional cell carcinoma/UC	842 (86.4)	554 (86.0)	123 (91.1)	29 (87.9)
Other	42 (4.3)	22 (3.4)	3 (2.2)	1 (3.0)
Comorbidities, *n* (%)	*n* = 378	*n* = 216	*n* = 50	*n* = 13
1	185 (48.9)	102 (47.2)	18 (36.0)	6 (46.2)
2	88 (23.3)	60 (27.7)	15 (30.0)	1 (7.7)
3	39 (10.3)	25 (11.6)	10 (20.0)	5 (38.5)
4+	66 (17.5)	29 (13.4)	7 (14.0)	1 (7.7)
Recorded deaths, *n* (%)	312 (32.0)	193 (30.0)	40 (29.6)	10 (30.3)
Data source, *n* (%)	*n* = 626	*n* = 415	*n* = 81	*n* = 24
Academic center	297 (47.4)	203 (48.9)	36 (44.4)	11 (45.8)
ASCO CancerLinQ	210 (33.5)	72 (17.3)	5 (6.2)	1 (4.2)
Community setting	119 (19.0)	140 (33.7)	40 (49.4)	12 (50.0)
Follow-up from aUC diagnosis, median (IQR), months	12 (12–13)	12 (11–14)	17 (15–20)	22 (21–27)
Follow-up from start of 1L PBC, median (IQR), months	10 (9–11)	10 (9–11)	13 (11–17)	20 (16–23)
Follow-up from start of avelumab 1L maintenance, median (IQR), months	NA	NA	9 (8–13)	15 (13–18)
Follow-up from start of 2L EV, median (IQR), months	NA	NA	NA	10 (6–14)

1L, first line; ASCO, American Society of Clinical Oncology; aUC, advanced urothelial carcinoma; EV, enfortumab vedotin; NA, not available; PBC, platinum-based chemotherapy.

**Table 2:** Real-world clinical outcomes in patients treated with 1L PBC and avelumab 1L maintenance.

Outcome	1L PBC (*n* = 644)	Avelumab 1L Maintenance (*n* = 135)
**Time on treatment, median (95% CI), months**	2.7 (2.5–3.0)	3.9 (2.8–5.0)
**OS**		
Median (95% CI), months	13.6 (12.1–17.8)	14.9 (13.1—NE)
6-month rate (95% CI), %	82 (78–87)	80 (72–90)
12-month rate (95% CI), %	56 (50–63)	63 (52–75)
18-month rate (95% CI), %	42 (36–50)	43 (31–59)
**PFS**		
Median (95% CI), months	3.5 (3.3–4.1)	6.4 (4.6—NE)
3-month rate (95% CI), %	65 (57–74)	73 (64–83)
6-month rate (95% CI), %	10 (6–17)	52 (42–65)
12-month rate (95% CI), %	2 (1–7)	40 (30–54)

Data in the 1L PBC and avelumab 1L maintenance subgroups were measured from the start of 1L PBC and the start of 1L maintenance, respectively. 1L, first line; NE, not estimable; OS, overall survival; PBC, platinum-based chemotherapy; PFS, progression-free survival.

## Data Availability

Deidentified data used in the research were collected in a real-world healthcare setting and are subject to controlled access for privacy and proprietary reasons. When possible, derived data supporting the findings of this study have been made available within the paper and its supplementary figures and tables.

## Supplementary Materials

The following supporting information can be downloaded at: https://www.mdpi.com/article/10.3390/curroncol33030138/s1. Figure S1: (a) Flow chart showing patient identification and (b) breakdown of 1L treatments received by patients who completed 1L treatment after the approval of avelumab 1L maintenance (July 2020 onward); Figure S2: (a) OS and (b) PFS from start of 2L EV in evaluable patients who received 2L EV after 1L PBC and avelumab 1L maintenance; Table S1: Definitions of outcomes; Table S2: Regimen details by treatment setting.

## References

[1] Siegel R.L., Miller K.D., Wagle N.S., Jemal A. (2023). Cancer statistics, 2023. CA Cancer J. Clin..

[2] National Cancer Institute (2023). Cancer Stat Facts: Bladder Cancer. https://seer.cancer.gov/statfacts/html/urinb.html.

[3] National Comprehensive Cancer Network (2025). NCCN Clinical Practice Guidelines in Oncology.

[4] Galsky M.D., Arija J.A.A., Bamias A., Davis I.D., De Santis M., Kikuchi E., Garcia-Del-Muro X., De Giorgi U., Mencinger M., Izumi K. (2020). Atezolizumab with or without chemotherapy in metastatic urothelial cancer (IMvigor130): A multicentre, randomised, placebo-controlled phase 3 trial. Lancet.

[5] Powles T., van der Heijden M.S., Castellano D., Galsky M.D., Loriot Y., Petrylak D.P., Ogawa O., Park S.H., Lee J.L., De Giorgi U. (2020). Durvalumab alone and durvalumab plus tremelimumab versus chemotherapy in previously untreated patients with unresectable, locally advanced or metastatic urothelial carcinoma (DANUBE): A randomised, open-label, multicentre, phase 3 trial. Lancet Oncol..

[6] Powles T., Csőszi T., Özgüroğlu M., Matsubara N., Géczi L., Cheng S.Y.S., Fradet Y., Oudard S., Vulsteke C., Morales Barrera R. (2021). Pembrolizumab alone or combined with chemotherapy versus chemotherapy as first-line therapy for advanced urothelial carcinoma (KEYNOTE-361): A randomised, open-label, phase 3 trial. Lancet Oncol..

[7] Powles T., Park S.H., Voog E., Caserta C., Valderrama B.P., Gurney H., Kalofonos H., Radulović S., Demey W., Ullén A. (2020). Avelumab maintenance therapy for advanced or metastatic urothelial carcinoma. N. Engl. J. Med..

[8] Powles T., Park S.H., Caserta C., Valderrama B.P., Gurney H., Ullén A., Loriot Y., Sridhar S.S., Sternberg C.N., Bellmunt J. (2023). Avelumab first-line maintenance for advanced urothelial carcinoma: Results from the JAVELIN Bladder 100 trial after ≥2 years of follow-up. J. Clin. Oncol..

[9] Padcev (enfortumab vedotin-ejfv) (2024). Prescribing Information.

[10] Opdivo (nivolumab) (2024). Prescribing Information.

[11] van der Heijden M.S., Sonpavde G., Powles T., Necchi A., Burotto M., Schenker M., Sade J.P., Bamias A., Beuzeboc P., Bedke J. (2023). Nivolumab plus gemcitabine–cisplatin in advanced urothelial carcinoma. N. Engl. J. Med..

[12] Powles T., Valderrama B.P., Gupta S., Bedke J., Kikuchi E., Hoffman-Censits J., Iyer G., Vulsteke C., Park S.H., Shin S.J. (2024). Enfortumab vedotin and pembrolizumab in untreated advanced urothelial cancer. N. Engl. J. Med..

[13] Fernandes L.E., Epstein C.G., Bobe A.M., Bell J.S.K., Stumpe M.C., Salazar M.E., Salahudeen A.A., Pe Benito R.A., McCarter C., Leibowitz B.D. (2021). Real-world evidence of diagnostic testing and treatment patterns in US patients with breast cancer with implications for treatment biomarkers from RNA sequencing data. Clin. Breast Cancer.

[14] Mo K., Wang X., Grad G., Scherrer E., Sangli C. (2025). RWD120 Comparison of demographics and clinical characteristics using real world data from Tempus multimodal database and SEER cancer registry across 17 solid cancer cohorts. Value Health.

[15] Kapilivsky J., Roth E., Rivers Z., Hockenberry A.J., Jain S., Warner J.L., Scherrer E., Sangli C. (2025). SA56 Assessing the completeness of oncology treatment data from administrative claims: A benchmarking study against abstracted EHRs using patient-level linkages. Value Health.

[16] Kapilivsky J., Roth E., Rivers Z., Hockenberry A.J., Jain S., Warner J.L., Cummings A.L., Grad G., Scherrer E., Sangli C. (2025). SA71 Integrating next generation sequencing, EHR, and claims data to extend follow-up in a real-world advanced lung adenocarcinoma biomarker-treatment landscape. Value Health.

[17] von Elm E., Altman D.G., Egger M., Pocock S.J., Gøtzsche P.C., Vandenbroucke J.P., STROBE Initiative (2007). Strengthening the Reporting of Observational Studies in Epidemiology (STROBE) statement: Guidelines for reporting observational studies. BMJ.

[18] Mamtani R., Zhang H., Parikh R.B., Patel K., Li H., Imai K., Hubbard R.A. (2023). Uptake of maintenance immunotherapy and changes in upstream treatment selection among patients with urothelial cancer. JAMA Netw. Open.

[19] Carroll C.E., Landrum M.B., Wright A.A., Keating N.L. (2023). Adoption of innovative therapies across oncology practices-evidence from immunotherapy. JAMA Oncol..

[20] Kearney M., Zhang L., Hubscher E., Musat M., Harricharan S., Wilke T. (2024). Undertreatment in patients with advanced urothelial cancer: Systematic literature review and meta-analysis. Future Oncol..

[21] Powles T., Park S.H., Voog E., Caserta C., Valderrama B.P., Gurney H., Kalofonos H., Radulovic S., Demey W., Ullén A. (2020). Maintenance avelumab + best supportive care (BSC) versus BSC alone after platinum-based first-line (1L) chemotherapy in advanced urothelial carcinoma (UC): JAVELIN Bladder 100 phase III interim analysis. J. Clin. Oncol..

[22] Barthélémy P., Loriot Y., Voog E., Eymard J.C., Ravaud A., Flechon A., Abraham Jaillon C., Chasseray M., Lorgis V., Hilgers W. (2023). Full analysis from AVENANCE: A real-world study of avelumab first-line (1L) maintenance treatment in patients (pts) with advanced urothelial carcinoma (aUC). J. Clin. Oncol..

[23] Grivas P., Barata P., Moon H., Gupta S., Hutson T., Sternberg C.N., Brown J.R., Dave V., Downey C., Shillington A.C. (2024). Avelumab first-line maintenance for locally advanced or metastatic urothelial carcinoma: Results from the real-world US PATRIOT-II study. Clin. Genitourin. Cancer.

[24] Antonuzzo L., Maruzzo M., De Giorgi U., Santini D., Tambaro R., Buti S., Carrozza F., Calabrò F., Di Lorenzo G., Fornarini G. (2024). READY: REAl-world Data from an Italian compassionate use program of avelumab first-line maintenance for locallY advanced or metastatic urothelial carcinoma. ESMO Real World Data Digit. Oncol..

[25] Bakaloudi D.R., Talukder R., Makrakis D., Agarwal N., Tripathi N., Bamias A., Zakopoulou R., Brown J., Korolewicz J., Fulgenzi C.A.M. (2023). Response and outcomes of maintenance avelumab after platinum-based chemotherapy (PBC) in patients (pts) with advanced urothelial carcinoma (aUC): “real world” experience. J. Clin. Oncol..

[26] Sridhar S.S., Powles T., Climent Durán M.A., Park S.H., Massari F., Thiery-Vuillemin A., Valderrama B.P., Ullén A., Tsuchiya N., Aragon-Ching J.B. (2024). Avelumab first-line maintenance for advanced urothelial carcinoma: Analysis from JAVELIN Bladder 100 by duration of first-line chemotherapy and interval before maintenance. Eur. Urol..

[27] Powles T., Rosenberg J.E., Sonpavde G.P., Loriot Y., Durán I., Lee J.L., Matsubara N., Vulsteke C., Castellano D., Wu C. (2021). Enfortumab vedotin in previously treated advanced urothelial carcinoma. N. Engl. J. Med..

[28] Nizam A., Jindal T., Jiang C.Y., Alhalabi O., Bakaloudi D.R., Talukder R., Davidsohn M.P., Nguyen C.B., Oh E., Taylor A.K. (2024). Outcomes in patients (pts) with advanced urothelial carcinoma (aUC) treated with enfortumab vedotin (EV) after switch maintenance avelumab (MAv) in the UNITE study. J. Clin. Oncol..

[29] Moon H.H., Kearney M., Mahmoudpour S.H., Ike C., Morris V., Rava A., Kim S., Sun H., Boyd M., Gomez Rey G. (2024). Real-world treatment patterns, sequencing, and outcomes in patients with locally advanced or metastatic urothelial carcinoma receiving avelumab first-line maintenance in the United States. Curr. Oncol..

[30] Fiala O., Massari F., Basso U., Giannatempo P., Grande E., Buti S., Myint Z.W., De Giorgi U., Pichler R., Grillone F. (2024). Enfortumab vedotin following platinum chemotherapy and avelumab maintenance in patients with metastatic urothelial carcinoma: A retrospective data from the ARON-2^EV^ study. Target Oncol..

[31] Barthélémy P., Thibault C., Fléchon A., Gross-Goupil M., Voog E., Eymard J.C., Abraham C., Chasseray M., Lorgis V., Hilgers W. (2025). Real-world study of avelumab first-line maintenance treatment in patients with advanced urothelial carcinoma in France: Overall results from the noninterventional AVENANCE study and analysis of outcomes by second-line treatment. Eur. Urol. Oncol..

[32] Benjamin D.J., Rezazadeh Kalebasty A., Prasad V. (2024). The overall survival benefit in EV-302: Is enfortumab vedotin plus pembrolizumab the new frontline standard of care for metastatic urothelial carcinoma?. Eur. Urol. Oncol..

