# Supplementary material for: Outcomes with Avelumab Maintenance Treatment for Advanced Urothelial Cancer in a US Patient Cohort
Source: Curr Oncol. 2026 Feb 27;33(3):138. doi: 10.3390/curroncol33030138 (PMC13025058; doi:10.3390/curroncol33030138)
Supplement: Supplementary file 1 [file curroncol-33-00138-s001.zip › Supplementary Figures S1 and S2.pdf]

Supplementary Figure S1. (a) Flow chart showing patient identification and (b) breakdown of 1L treatments received by patients who completed 1L treatment after the approval of avelumab 1L maintenance (July 2020 onward)

(a)

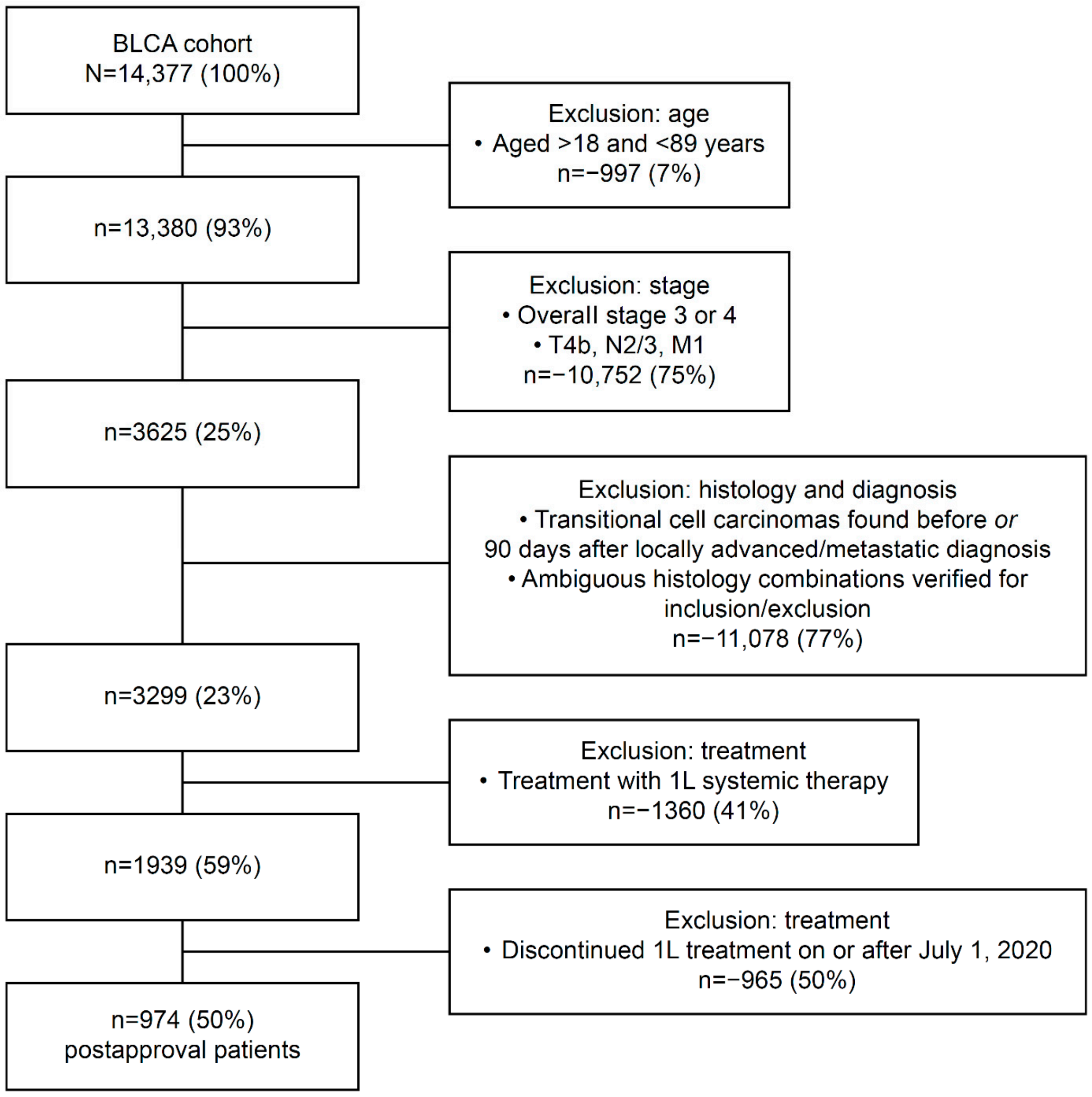

(b)

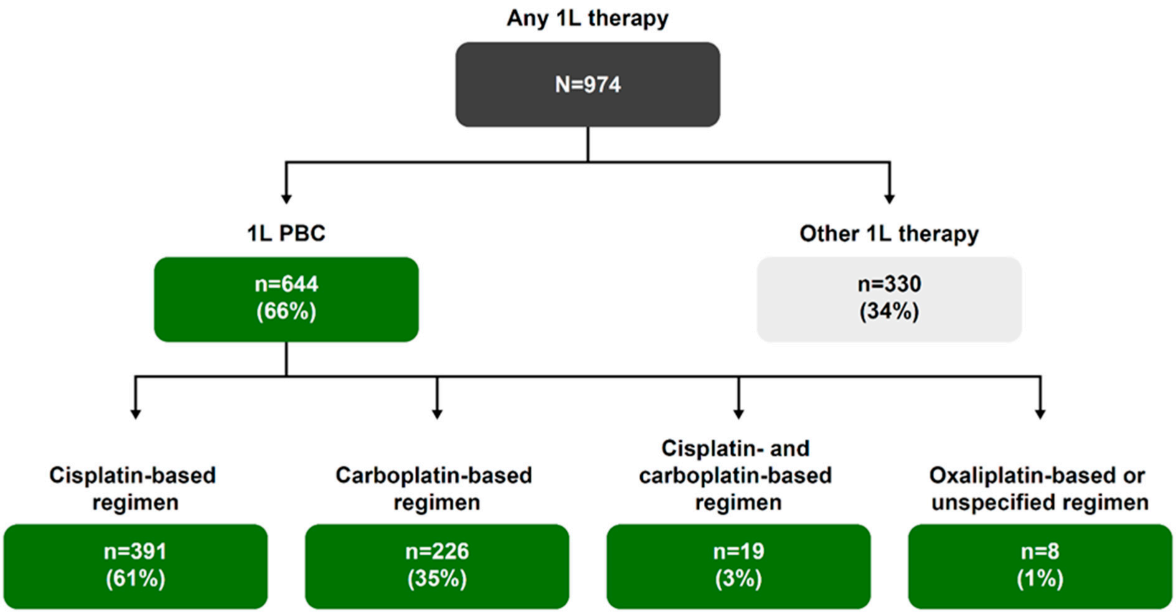

1L, first line; BLCA, bladder cancer; PBC, platinum-based chemotherapy.

Supplementary Figure S2. (a) OS and (b) PFS from start of 2L EV in evaluable patients who received 2L EV after 1L PBC and avelumab 1L maintenance

(a)

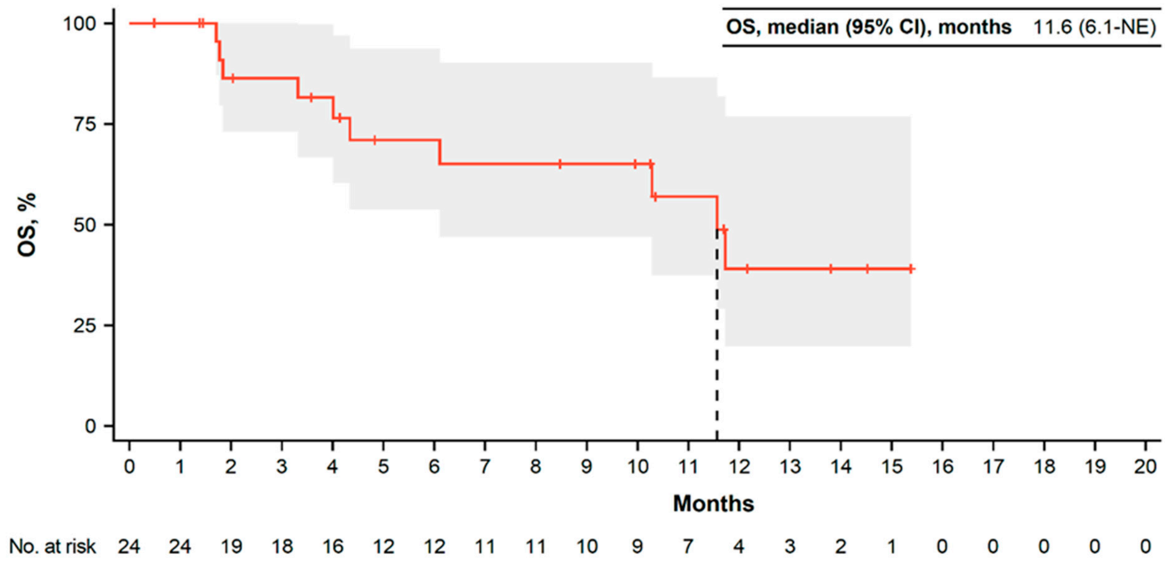

(b)

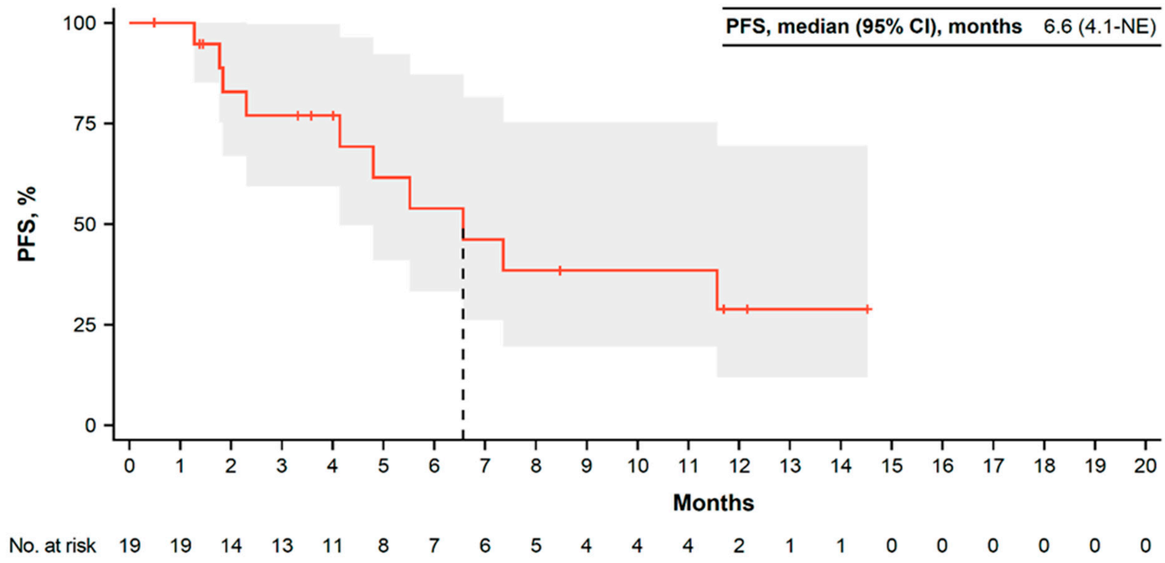

Patients were not included if dates of progression or 2L EV treatment initiation were missing.
1L, first line; 2L, second line; EV, enfortumab vedotin; NE, not estimable; OS, overall survival; PBC, platinum-based chemotherapy; PFS, progression-free survival.
