# Supplementary material for: Outcomes with Avelumab Maintenance Treatment for Advanced Urothelial Cancer in a US Patient Cohort
Source: Curr Oncol. 2026 Feb 27;33(3):138. doi: 10.3390/curroncol33030138 (PMC13025058; doi:10.3390/curroncol33030138)
Supplement: Supplementary file 1 [file curroncol-33-00138-s001.zip › Supplementary Tables S1 and S2.pdf]

Supplementary Table S1. Definitions of outcomes

Outcome	Definition
Complete response	Complete resolution of all visible disease in response to treatment; assessed pathologically or radiologically
Partial response	Partial reduction in size of visible disease or extent of disease; assessed pathologically, radiologically, or biochemically, or by physical examination
Stable disease	No change in overall volume of visible disease
Progressive disease	Increase in visible disease, increase in extent of disease, or identification of new lesions; assessed pathologically or radiologically and dictated by the physician

Supplementary Table S2. Regimen details by treatment setting

Treatment setting	Patients, n/N (%)	Regimen, n (%)
**1L**	974 (100)	Cisplatin plus gemcitabine, 297 (30.5)<br>Carboplatin plus gemcitabine, 200 (20.5)<br>Pembrolizumab, 154 (15.8)<br>MVAC, 60 (6.2)<br>Nivolumab, 36 (3.7)<br>Other, 227 (23.3)
**1L maintenance**	219/644 (34.0)[a]	Avelumab, 135 (61.6)<br>Pembrolizumab, 63 (28.8)<br>Nivolumab, 11 (5.0)<br>Atezolizumab, 5 (2.3)<br>Durvalumab, 2 (0.9)<br>Other ICI, 3 (1.4)
**2L**	258/974 (26.5)[b]	Enfortumab vedotin, 70 (27.1)<br>Pembrolizumab, 47 (18.2)<br>Carboplatin plus gemcitabine, 34 (13.2)<br>Cisplatin plus gemcitabine, 17 (6.6)<br>Nivolumab, 17 (6.6)<br>Erdafitinib, 10 (3.9)<br>Gemcitabine, 10 (3.9)<br>Other, 53 (20.5)
**3L**	74/258 (28.7)[c]	Enfortumab vedotin, 15 (20.3)<br>Pembrolizumab, 10 (13.5)<br>Avelumab, 10 (13.5)

		Erdafitinib, 9 (12.2)<br><br>Sacituzumab govitecan, 7 (9.5)<br><br>Atezolizumab, 4 (5.4)<br><br>Carboplatin plus gemcitabine, 4 (5.4)<br><br>Other, 15 (20.3)

1L, first line; 2L, second line; 3L, third line; ICI, immune checkpoint inhibitor; MVAC, methotrexate, vinblastine, doxorubicin, and cisplatin.
[a] Percentage of patients who received 1L platinum-based chemotherapy.
[b] Percentage of patients who received any 1L treatment.
[c] Percentage of patients who received 2L treatment.
